# Combining convolutional attention mechanism and residual deformable Transformer for infarct segmentation from CT scans of acute ischemic stroke patients

**DOI:** 10.3389/fneur.2023.1178637

**Published:** 2023-07-20

**Authors:** Zhixiang Xu, Changsong Ding

**Affiliations:** ^1^School of Informatics, Hunan University of Chinese Medicine, Changsha, Hunan, China; ^2^Big Data Analysis Laboratory of Traditional Chinese Medicine, Hunan University of Chinese Medicine, Changsha, Hunan, China

**Keywords:** Transformer, convolutional attention mechanism, infarct segmentation, volumetric analysis, acute ischemic stroke, non-contrast CT

## Abstract

**Background:**

Segmentation and evaluation of infarcts on medical images are essential for diagnosis and prognosis of acute ischemic stroke (AIS). Computed tomography (CT) is the first-choice examination for patients with AIS.

**Methods:**

To accurately segment infarcts from the CT images of patients with AIS, we proposed an automated segmentation method combining the convolutional attention mechanism and residual Deformable Transformer in this article. The method used the encoder-decoder structure, where the encoders were employed for downsampling to obtain the feature of the images and the decoder was used for upsampling and segmentation. In addition, we further applied the convolutional attention mechanism and residual network structure to improve the effectiveness of feature extraction. Our code is available at: https://github.com/XZhiXiang/AIS-segmentation/tree/master.

**Results:**

The proposed method was assessed on a public dataset containing 397 non-contrast CT (NCCT) images of AIS patients (AISD dataset). The symptom onset to CT time was less than 24 h. The experimental results illustrate that this work had a Dice coefficient (DC) of 58.66% for AIS infarct segmentation, which outperforms several existing methods. Furthermore, volumetric analysis of infarcts indicated a strong correlation (Pearson correlation coefficient = 0.948) between the AIS infarct volume obtained by the proposed method and manual segmentation.

**Conclusion:**

The strong correlation between the infarct segmentation obtained via our method and the ground truth allows us to conclude that our method could accurately segment infarcts from NCCT images.

## 1. Introduction

Stroke refers to sudden brain dysfunction caused by cerebral blood circulation disorder and is one of the most prevalent fatal illnesses. Stroke can be grouped into two types: ischemic and hemorrhagic stroke. These are caused by blockage or rupture of cerebral blood vessels, respectively. Most patients with stroke suffer from acute ischemic stroke (AIS) (1). Due to the fast speed and low expense of CT, it has become the first-choice imaging technique for diagnosis and prognosis of stroke (2). In addition, the comparative analysis of the infarct volume in non-contrast CT (NCCT) images before and after treatment can facilitate judgment of the effectiveness of treatment. NCCT imaging is one of the essential methods for AIS diagnosis (3), and rapid segmentation of infarcts is crucial for AIS diagnosis. Manual segmentation is mainly utilized in clinical practice to ensure segmentation accuracy. However, problems exist with this method, such as excessive reliance on prior knowledge in the medical field and human evaluation errors (4). Therefore, the segmentation of medical images can be challenging in medical image analysis (5). Many methods have treated the segmentation of AIS infarcts as an anomaly detection task, determining the differences between the infarct and surrounding tissues (6). Nevertheless, detecting the infarct can only provide a rough assessment of AIS, which cannot effectively guide diagnosis or the development of corresponding treatment plans. To segment AIS infarcts from medical images, several machine learning (ML)-based (7) segmentation methods, such as SVM and random forest (8), have been proposed. For example, Kuang et al. (9) proposed a convex optimization method based on random forest classification.

In order to enhance the segmentation performance, segmentation methods based on deep learning (DL) (10) have been proposed. The CNN-based AIS infarct segmentation method achieved impressive performance (11). A full convolutional neural network (FCN) removed the fully connected layers in CNN and elevated image segmentation from image-level to pixel-level. Zhang et al. (12) combined 3D FCN with dense connections to automatically segment AIS infarcts. Compared to ordinary images, medical images have a wide grayscale range and unclear boundaries. To address these issues, U-Net (13) based on FCN was proposed. U-Net employs an encoder-decoder architecture with skip connections between downsampled and upsampled information to improve segmentation accuracy. U-Net has been refined through advancements such as U-Net++ (14) and Res-UNet (15), and these improvements have yielded noteworthy outcomes in numerous image processing domains. Ni et al. (16) proposed a novel asymmetry disentanglement network (ADN), where asymmetric disentanglement of the input NCCTs is first conducted to produce various 3D asymmetric maps. Subsequently, a synthesized intrinsic asymmetry-compensated pathologically enhanced NCCT volume is created and utilized as the input for the segmentation network to segment the AIS infarct, achieving good performance. The backbone of ADN is 3D ResidualUnet, which leverages convolutions as the fundamental operations to perform feature extraction and encoding on the NCCT. However, the limited receptive field of convolutional operations constrains their ability to capture global dependencies (17). Therefore, many studies have explored the use of Transformers in image analysis, which are known for their strong global modeling capabilities. A typical example is the Vision Transformer (ViT) (18), which utilizes a pure Transformer structure to process image patches and demonstrated remarkable achievements in image recognition tasks. SETR (19) employs a Transformer as the encoder and a CNN architecture as the decoder to create a superior-performing segmentation model. The encoder in TransUNet (20) connects CNN with the Transformer and employs the latter to process the final layer features produced by the CNN, resulting in remarkable performance and effectiveness. However, the complexity of training a Transformer for image tasks is considerable and requires high-performance computers.

Therefore, to solve the above problems, we aimed to develop an automatic segmentation method in this work to accurately segment infarcts from NCCT images of AIS patients. Our method is distinguished by the following four characteristics: (1) by combining CNN and a Transformer, we optimize their performance by mitigating their limitations and enhancing their advantages; (2) to improve the efficiency of local feature extraction, the convolutional block attention module (CBAM) is used to direct attention to the key areas of segmentation; (3) to simplify the Transformer for image tasks and to improve the efficiency of model training, deformable multi-heads self-attention (DMSA) is introduced to distribute attention to a few key points around the sampling points instead of to all points in the feature map; (4) given the information loss during transmission via the Transformer layer, we integrate a residual connection before and after the Transformer encoder to enhance the information and achieve better segmentation.

## 2. Materials and methods

We conducted the experiment on the AIS dataset (AISD) (21) in this study. It consists of 397 NCCT scans of acute ischemic strokes acquired within 24 h of the patient's symptom onset. In addition, patients had a diffusion-weighted MRI (DWI) within a day of receiving the CT scan. The NCCT scans had a slice thickness of 5 mm. Labels were manually annotated by a doctor and carefully checked by another senior doctor. According to the data division in the original article, 345 scans of patients were employed for model training and parameter tuning, and the remaining 52 NCCT scans were employed for evaluating the proposed method.

We employed the *Z*-score method to normalize the contextual feature information of the original NCCT dataset. Image normalization can optimize the efficiency of DL in segmentation tasks (22). To avoid overfitting problems caused by the limited training data, we used data augmentation methods to diversify it. The methods used include random rotation, Flip, zoom, adding Gaussian white noise, Gaussian blur, adjusting accuracy and contrast, gamma transform, and simulating low resolution.

**Figure 1:**
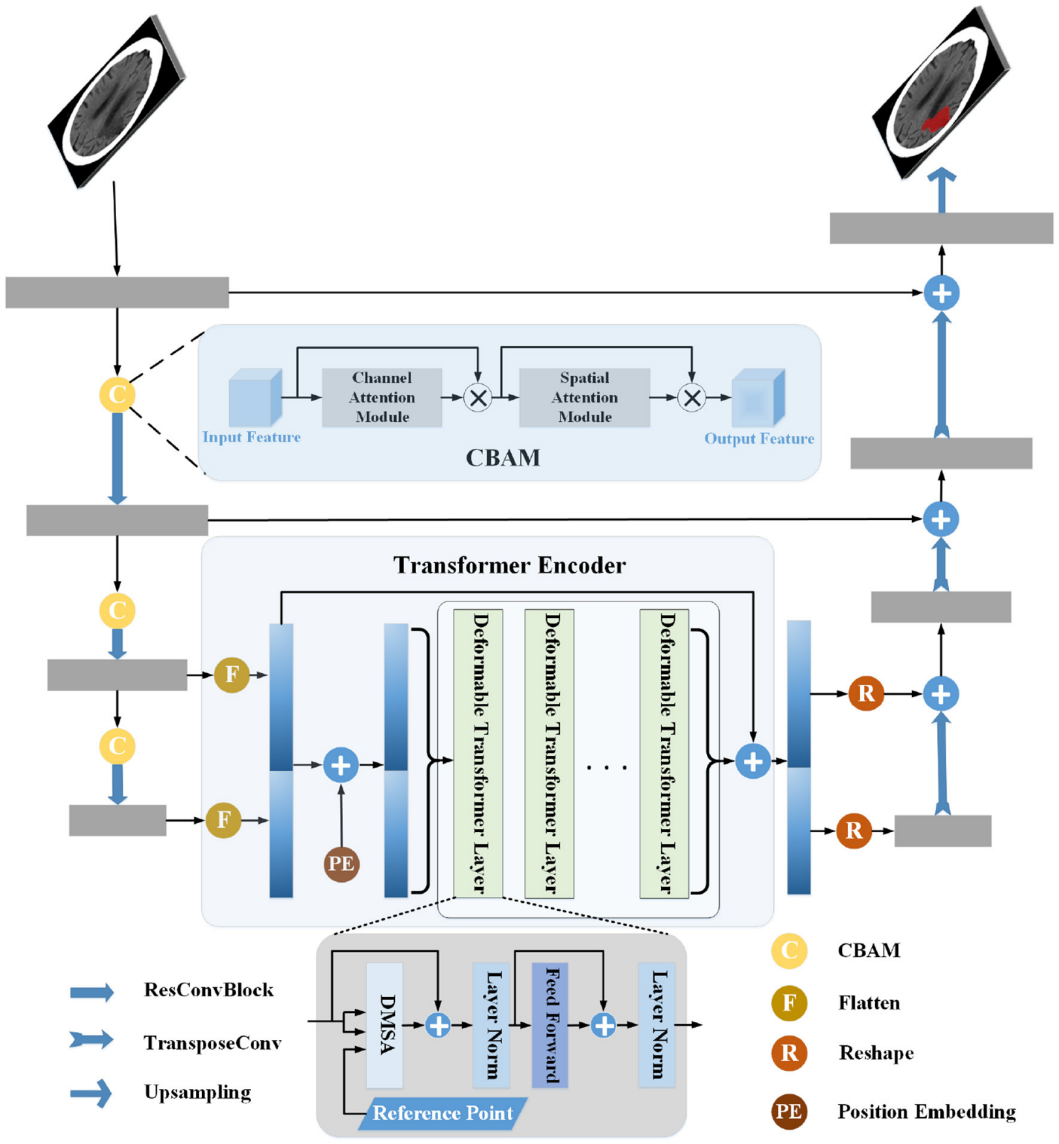
Pipeline of the proposed infarct segmentation method. The structure of the CNN Encoder is shown in the left part of the figure, the middle part shows the Transformer Encoder, and the right part shows the structure of the Decoder. The gray rectangle in the figure is the CNN block, the yellow circle is the convolutional block attention module, and the green rectangle is the Deformable Transformer layer. The CNN Encoder extracts the image's local features, connects the last two layers of features, and passes them into the Transformer Encoder for global relationship modeling. Then, the local and global features are sent to the decoder for processing and segmentation.

The segmentation method proposed in this work consisted of two encoders and a decoder, including a CNN Encoder, Transformer Encoder, and Decoder. The encoders obtained the features, and the feature maps were passed to the Decoder for upsampling. They were restored to the same resolution as the source image and segmentation was finally achieved. The CNN Encoder extracted the local features of the image and then modeled global dependency on them via the Transformer Encoder. In addition, the CNN Encoder used CBAM to direct attention to the feature maps. In the Transformer Encoder, DMSA was used to make it simpler, and a residual connection was employed before and after the Transformer Encoder to enhance the information. The structure is shown in Figure 1.

### 2.1. CNN Encoder

The CNN Encoder encoded the input image with multiple convolutional layers, similar to convolutional pyramids. It extracted the local features of the image, in which the CBAM (23) was used to compute effective local attention maps, strengthen the influence of the infarct area, and reduce feature redundancy for segmentation.

The CNN Encoder contained a Conv-IN-ReLU block and three residual convolutional blocks (ResConvBlock). The Conv-IN-ReLU block comprised a convolutional layer with a large kernel and followed processes with instance normalization (IN) (22) and ReLU activation. The intermediate feature map was obtained after the Conv-IN-ReLU block. The given input image was *x*∈*R*^*C*×*D*×*H*×*W*^. *D*, *H*, *W*, and *C* individually represented depth, height, width, and channels. After each ResConvBlock, *D*, *H*, and *W* were halved. The ResConvBlock downsampled the upper-layer feature maps to high-level and coarse-resolution. The CBAM was employed to integrate channel and spatial attention at local levels, allowing the CNN encoder to effectively capture significant features and their locations, leading to improved performance.

**Figure 2:**
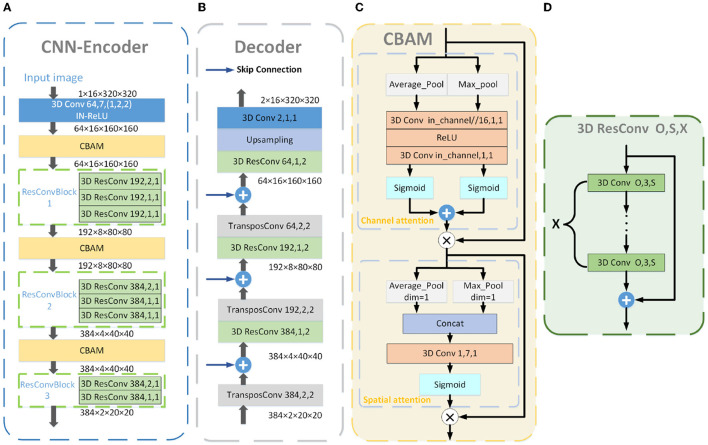
Detailed structure blueprint of the CNN Encoder **(A)**, Decoder **(B)**, and CBAM **(C)**. The blue “3D Conv” in the CNN Encoder consists of a 3D convolutional layer and processes with IN and ReLu activation. The detailed structure of the yellow “CBAM” is shown in **(C)**, consisting of the CAM and SAM. The specific structure of the green “3D ResConv” is shown in **(D)**. The orange “3D Conv” is a 3D convolutional layer. The gray “TransposeConv” is a 3D transposed convolutional layer. The blue arrows represent the skip connections. The numbers in the convolutional blocks indicate the convolution kernels and stride, and those in the residual convolutional blocks denote the convolution kernels, stride, and convolutional layers.

The detailed layout of the CNN Encoder is shown in Figure 2A and contains a Conv-IN-ReLU block, three CBAM blocks, and three ResConvBlock, which consisted of three, three, and two 3D residual convolution operations, respectively. The specific structure of ResConvBlock is shown in Figure 2D.

### 2.2. CBAM

CBAM consisted of a channel attention module (CAM), followed by a spatial attention module (SAM; as illustrated in Figure 2C). Channels can be deemed to be feature detectors in feature maps so that the CAM can pay attention to features with greater importance. The intermediate feature map served as the input for the CAM, with feature information aggregated along the spatial axis using global average pooling and max pooling. Following that, the two pooled features were forwarded to a shared feed-forward network, resulting in a 1D channel attention map. It can be formulated as follows:


(1) $$\begin{array}{l} \begin{array}{c} CAM\left(M\right)=\sigma \left(MLP\left(AP\left(M\right)\right)+MLP\left(MP\left(M\right)\right)\right)\\ =\sigma \left(K_{1}\left(K_{0}\left(M_{avg}^{c}\right)\right)+K_{1}\left(K_{0}\left(M_{max}^{c}\right)\right)\right), \end{array} \end{array}$$

where *M* is the intermediate feature map, AP and MP represent the average and max pooling, $M_{avg}^{c}$ and $M_{max}^{c}$ represent two pooled features on the spatial axis, respectively, and *CAM*(*M*) denotes the obtained channel attention map.

The channel attention map was fused with the intermediate feature map by element-wise multiplication, and the formulation is as follows:


(2) $$\begin{array}{l} M^{\prime }=CAM\left(M\right)⊗M, \end{array}$$

where ⊗ represents element-wise product and *M*′ is the feature map with channel attention.

The SAM used *M*′ as the input. Similar to the operations of the CAM, *M*′ was aggregated in the direction of the channel axis using average-pooling and max-pooling operations, which can emphasize the location information of essential features (24). Then, the two-pooled information was concatenated and a spatial attention map in 2D was produced using a convolution operation. The formulation of this is as follows:


(3) $$\begin{array}{l} SAM(M^{\prime })=\sigma (k_{7\times 7}([AP(M^{\prime }));MP(M^{\prime })]))\\ \;=\sigma (k_{7\times 7}([M_{avg}^{s};M_{max}^{s}])), \end{array}$$

where $M_{avg}^{S}$ and $M_{max}^{S}$ represent the two pooled features on the channel axis, and the generated spatial attention map is denoted by *SAM*(*M*′).

The *SAM*(*M*′) was fused with the obtained features of CAM to focus on essential features and locations. The formulation is as follows:


(4) $$\begin{array}{l} M^{\prime \prime }=SAM\left(M^{\prime }\right)⊗M^{\prime }, \end{array}$$

where *M*″ represents the final refined output.

### 2.3. Transformer Encoder

Since the receptive field of CNN was limited, it was difficult for convolution operations to capture the global dependency of the feature map (17); therefore, a Transformer Encoder was introduced. The Transformer achieved outstanding results in natural language processing. Its self-attention could obtain global dependency, making each word pay attention to other words at all positions in the sentence. However, in image tasks, slow convergence and high computational complexity would result if each point focused on other points in all positions. We used deformable self-attention (DSA) (25) to concentrate on a limited number of key points around each reference point to solve this problem and to improve model efficiency.

The results of the CNN Encoder served as the source information for the Transformer encoder. Since the Transformer is a sequential model, we need to convert the feature maps into a sequence. However, the process of feature serialization would cause the loss of position information; therefore, we used position embedding in the Transformer to complete the 3D position information. The following formula obtains the position embedding:


(5) $$\left\{ \begin{array}{l} \;Em_{\left\{D,H,W\right\}}(P,2k)=sin(P\cdot w)\\ Em_{\left\{D,H,W\right\}}(P,2k+1)=cos(P\cdot w), \end{array}\right.$$

using the sine and cosine functions alternately to obtain the position embedding, where {*D, H, W*} represent different dimensions, *Em*_*D*_, *Em*_*H*_, and *Em*_*W*_ form the 3D position embedding of the position *P* with *k* dimension, and $w=1/10,000^{2k/\frac{C}{3}}$. We added the position embedding to the serialized CNN encoding using the corresponding element summation method before being fed into the Transformer Encoder.

The Transformer Encoder used the multi-layer features of the CNN encoder, and the position embedding was different at the same position of each layer. Therefore, *L* represents the layers of the feature map, and *f*_*l*_ denotes features in the *l*-th layer. *Z*_*q*_ is the feature of the query *q*, and *p*_*q*_ represents the 3D position of the reference point; thus, the DMSA can be formulated as:


(6) $$\begin{array}{l} DeformAttn=\overset{M}{\underset{m}{\sum }}W_{m}\left\{\overset{L}{\underset{l}{\sum }}\overset{K}{\underset{k}{\sum }}A_{mlqk}•W_{m}^{\prime }f_{l}\left[\sigma \left(p_{q}\right)+\Delta p_{mlqk}\right]\right\}, \end{array}$$

where *M* is the number of heads of the self-attention, *K* represents the quantity of key sampled points, σ(•) denotes the Sigmoid function that adjusts *p*_*q*_ to the feature of *l*-th level, Δ*p*_*mlqk*_ is the offset of the sampled point, and *A*_*mlqk*_ represents the attention weight and is in the range of [0, 1].

The Transformer Encoder consisted of six stacked Deformable Transformer layers. One Deformable Transformer layer contained a DMSA layer and two normalization operations, followed by a feed-forward network. In addition, the input of the Transformer Encoder was residually added to the output to enhance the local information and to compensate for the loss caused by the transmission of information in the Transformer Encoder.

### 2.4. Decoder

The Decoder used the pure transposed convolution operations to restore the encoded feature maps and gradually upsampled them to *D*×*H*×*W*, the same as the input size. The output sequence was re-formed into the feature maps. After that, the transposed convolution operations were performed. The skip-connection between a certain level of the CNN-Encoder and the corresponding level of the Decoder added fine details to make the segmentation more accurate. The 3D residual convolutions were utilized to enhance the upsampled feature maps.

Figure 2B illustrates the Decoder's detailed structure, which contains four upsampling blocks. In the first three up-sampling blocks, the feature map was subjected to a transposed convolution operation and refined by a 3D residual convolution operation. The Decoder was skip-connected with the CNN Encoder. The last block contained an upsampling layer and a 3D convolutional with a kernel size of 1–1, which mapped the features to the number of categories for classification.

### 2.5. Loss function

Dice loss (26) was used to evaluate the correlation between two regions. It shows outstanding performance when the positive and negative samples are unequal in the data. CrossEntropy loss was used to find the overall average loss. We combined the Dice loss and CrossEntorpy loss (27) in our model, calculated as follows:


(7) $$\begin{array}{l} \begin{array}{c} \varepsilon \left(Y,P\right)=Dice\text{\_}loss+CrossEntropy\text{\_}loss\\ =-\frac{1}{N}\overset{C}{\underset{c=1}{\sum }}\overset{N}{\underset{n=1}{\sum }}\left(y_{n,c}logp_{n,c}+\frac{2y_{n,c}p_{n,c}}{y_{n,c}^{2}p_{n,c}^{2}}\right), \end{array} \end{array}$$

where *Y*, *P*, *C*, and *N* represent the infarct ground truth (GT), the infarcts segmented by the proposed module results, the number of categories, and the number of pixels, respectively.

### 2.6. Implementation details

During the model training phase, we randomly cropped patches with a size of 16 × 32 × 320 from NCCT scans as input images. The model underwent 350 epochs of training and 250 iterations per epoch. The learning rate was configured to 0.001, the stochastic gradient descent method was used to adjust it, and the momentum value was assigned to 0.99. According to the experiments, we set the number of 3D residual convolution layers contained in the three ResConvBlock stages to three, three, and two, the key points (K) to four, the heads (H) to six, and the number of layers in the Deformable Transformer (L) to six. The implementation and evaluation of our method were conducted on a server equipped with an NVIDIA A40 GPU.

The sliding window approach was employed for testing, and the window size was the same as the patch size in the training phase. To evaluate the effects of all compared infarct segmentation methods, we calculated the Dice score to assess the regional correlation between them; the range of possible values for the Dice score is [0, 1]. Furthermore, we calculated the F1-score, Recall, and Precision to evaluate the infarct level.

## 3. Results

### 3.1. Comparison with existed methods

**Figure 3:**
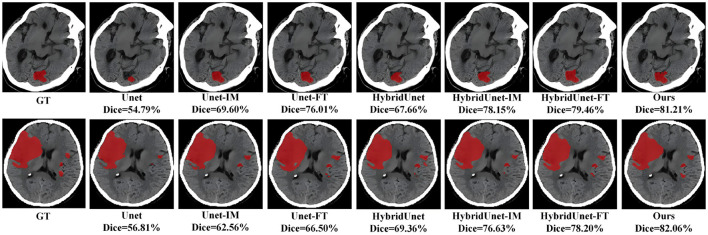
Visual qualitative comparisons of the six compared methods with the proposed method for two AIS cases.

We contrast the proposed method with several already existing methods, as follows: Unet (13) using a CNN architecture purely, an image-level method (Unet-IM) (28), a feature-level method (Unet-FT) (29), a method using a 3D convolutional block as the basic encoding block (HybridUnet) (30) and its implementation at the image-level, pixel-level (HybridUnet-IM, HybridUnet-FT), and a Symmetric Enhanced Attention Network (SEAN) (21). Table 1 presents the number of parameters, running time, and segmentation effectiveness of these methods. It can be observed that the Dice score and recall of our method are 58.66% and 0.6319, respectively, which are better than the existing methods. In addition, our method produces better results than the compared methods in terms of the F1-score (0.6298), although our method did not achieve the best result on precision. Since our method jointly considers the local features and global relationships of images, it could better process the detailed and overall information of the image so that the segmentation results are more similar to the GT. There was an increase in the number of parameters and running time for our method because of the Transformer encoder; however, the use of deformable self-attention rendered this increase within an acceptable range. Moreover, our method achieved the best segmentation performance. Two segmentation examples, the Dice score of the proposed method, and six compared methods for the AISD infarct segmentation task are shown in Figure 3.

**Table 1:** Quantitative comparison of our proposed method with seven baselines for AIS infarct segmentation, where M = 1,024 – 1,024, h represents hours, and s represents seconds.

**Method**	**Params (M)**	**Train time (h)**	**Test time (s)**	**Dice (%)**	**F1 score**	**Recall**	**Precision**
Unet	39.57	8.7	11.5	45.88	0.5105	0.5019	0.5196
Unet-IM	40.14	13.3	13.8	50.35	0.5457	0.5318	0.5603
Unet-FT	44.85	19.4	17.3	53.54	0.5720	0.5655	0.5786
HybridUnet	45.04	22.6	25.4	49.52	0.5433	0.6105	0.4895
HybridUnet-IM	46.61	24.7	25.4	54.37	0.5992	0.5581	0.6471
HybridUnet-FT	50.32	26.9	30.0	55.77	0.6015	0.5431	**0.6742**
SEAN	-	-	-	57.84	0.6218	0.5880	0.6597
**Ours**	58.62	30.9	35.8	**58.66**	**0.6298**	**0.6319**	0.6278

The bold values indicate the optimal performance of the metric in the experiment.

To statistically analyze the AIS infarct segmentation results, we conducted pair-wise comparisons (based on the Dice score) using the Wilcoxon rank-sum test between the proposed method and the six compared methods. When the *p*-value < 0.05, this suggests a statistically significant difference between the two methods being tested. Table 2 presents the results of the statistical analysis, which demonstrate that the proposed method outperformed the six compared methods in AIS infarct segmentation. The differences were statistically significant (all *p*-values < 0.05). Thus, the proposed method significantly improved the segmentation of AIS infarcts.

**Table 2:** Statistical test results (*p*-value) between the proposed method and six compared methods.

**Metrics**	***p*-value**
Unet	2.27 × 10^−8^
Unet-IM	4.68 × 10^−5^
Unet-FT	1.73 × 10^−3^
HybridUnet	1.34 × 10^−5^
HybridUnet-IM	1.69 × 10^−2^
HybridUnet-FT	1.79 × 10^−2^

To evaluate the effectiveness and generalizability of our method on different datasets, we also compared our method with six other methods for infarct segmentation using the ISLES2018 (31, 32) training set. The ISLES2018 training dataset consists of multi-modal CT image data from 94 ischemic stroke patients, of which we only used CT and OT data. We trained and tested the method using five-fold cross-validation. As shown in Table 3, our method achieved a Dice score of 46.67%, an F1 score of 0.5242, a recall of 0.4724, and a precision of 0.5888, all of which outperformed the other methods and effectively segmented the infarcts, demonstrating the generality of our method on other datasets.

**Table 3:** Quantitative results for evaluation of the ISLES2018 dataset.

**Method**	**Dice (%)**	**F1 score**	**Recall**	**Precision**
Unet	35.94	0.3816	0.2871	0.5689
Unet-IM	38.03	0.4419	0.35801	0.5771
UNet-FT	40.59	0.4802	0.4150	0.5698
HybridUNet	41.81	0.4944	0.4326	0.5769
HybridUNet-IM	42.88	0.4990	0.4382	0.5795
HybridUNet-FT	4324	0.5065	0.4688	0.5509
**Ours**	**46.67**	**0.5242**	**0.4724**	**0.5888**

The bold values indicate the optimal performance of the metric in the experiment.

### 3.2. Volumetric analysis

We calculated the AIS infarct volume segmented based on the proposed method (*V*_*S*_) and the manually segmented infarct volume (*V*_*M*_). Pearson correlation calculations of *V*_*S*_ with *V*_*M*_ were performed and Bland-Altman plots were used to analyze the volumes. Furthermore, we calculated the volume difference between *V*_*S*_ and *V*_*M*_: Δ*V*_*diff*_ = *V*_*S*_−*V*_*M*_ and the absolute volume difference: |Δ*V*_*diff*_| = |*V*_*S*_−*V*_*M*_|. Additionally, to show the clinical relevance of our results, we performed dichotomization, with 70 cc as the cut-off value in our analysis of the AIS infarct volume. We evaluated the dichotomization analysis using Accuracy, Kappa, and Specificity as metrics.

**Figure 4:**
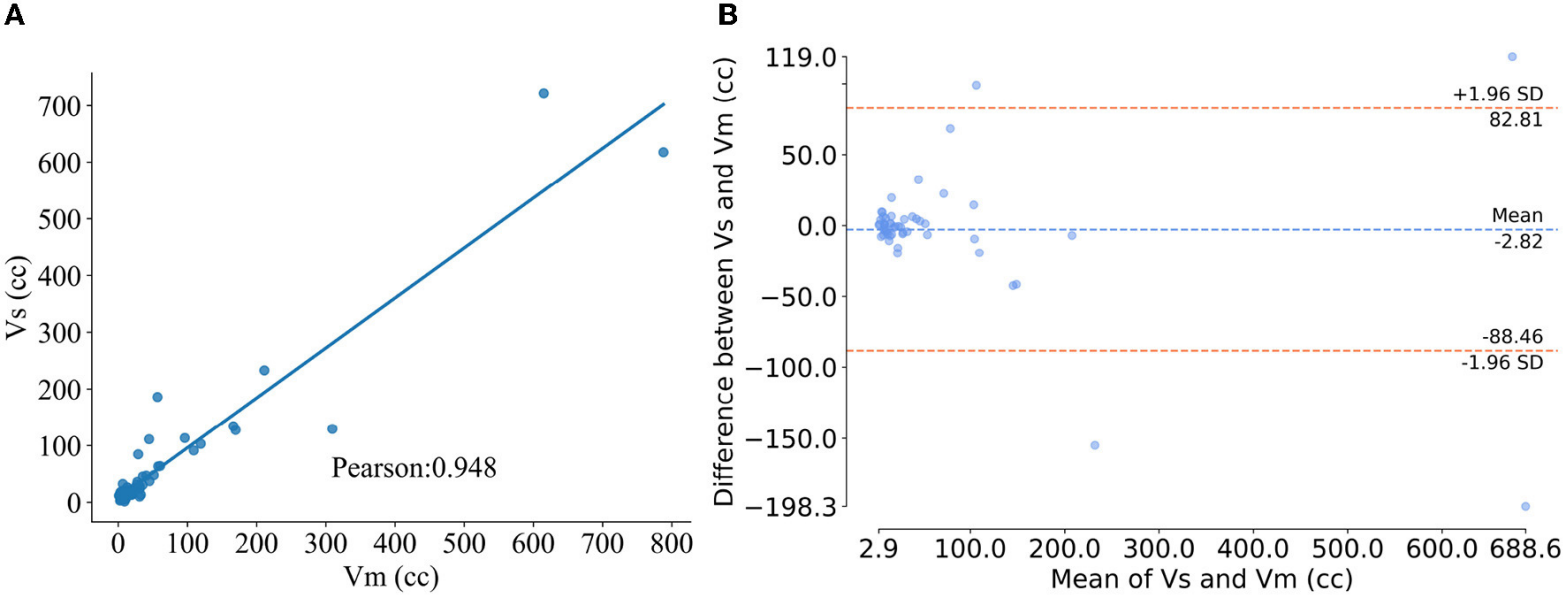
Results of volumetric analysis. **(A)** Pearson correlation plot of method segmentation versus manual segmentation of the infarct volume, where the abscissa and ordinate represent the infarct volume segmented manually and using the proposed method, respectively. **(B)** Blad-Altman plot for the two segmentation methods, where the x-axis is the average of VS and VM and the y-axis is the Δ*Vdiff*.

Table 4 displays the volumetric analysis results. The average volume difference between *V*_*S*_ and *V*_*M*_ is 2.82 cc and the absolute average volume difference is 19.86. The volume correlation between *V*_*S*_ and *V*_*M*_ is r=0.948 (95% confidence interval: 0.916–0.972, *p* < 0.001), reflecting the excellent correlation between them, see Figure 4A. The average volume difference between *V*_*S*_ and *V*_*M*_ is shown in the Bland-Altman diagram in Figure 4B, from which it could be seen that they have good consistency. The excellent correlation and consistency of *V*_*S*_ and *V*_*M*_ verify the utility of our method. Table 5 illustrates the results of the dichotomization analysis, with the cut-off set at 70 cc, for our method and six other compared methods. We can see from Table 3 that our method achieves an accuracy of 96.2% (95% confidence interval: 93.4–97.8), a Kappa of 0.88 (95% confidence interval: 0.79–0.93), and a Specificity of 0.95 (95% confidence interval: 0.92–0.97), surpassing the performance of other methods.

**Table 4:** Volumetric analysis results between *V*_*S*_ and *V*_*M*_.

**Metrics**	**Result**
Δ*V*_*diff*_(cc)	−2.82 ± 44.12
|Δ*V*_*diff*_|(cc)	19.86 ± 39.40
Correlation	0.948

Values are represented as the mean ± one standard deviation.

**Table 5:** Classification performance when using 70 cc as the infarct volume cut-off (95% confidence interval is shown in parentheses).

**Methods**	**Accuracy (%)**	**Kappa**	**Specificity**
Unet	86.5 (77.6–92.1)	0.64 (0.45–0.78)	0.84 (0.73–0.90)
Unet-IM	88.4 (80.6–93.2)	0.68 (0.50–0.80)	0.86 (0.77–0.92)
Unet-FT	90.4 (83.8–94.4)	0.72 (0.56–0.83)	0.88 (0.80–0.93)
HybridUnet	88.2 (80.2–93.1)	0.67 (0.49–0.80)	0.85 (0.76–0.91)
HybridUnet-IM	92.3 (86.9–95.5)	0.77 (0.63–0.86)	0.91 (0.85–0.95)
HybridUnet-FT	94.2 (90.1–96.7)	0.82 (0.71–0.89)	0.93 (0.88–0.96)
**Ours**	**96.2 (93.4–97.8)**	**0.88 (0.79–0.93)**	**0.95 (0.92–0.97)**

The bold values indicate the optimal performance of the metric in the experiment.

### 3.3. Ablation study

**Figure 5:**
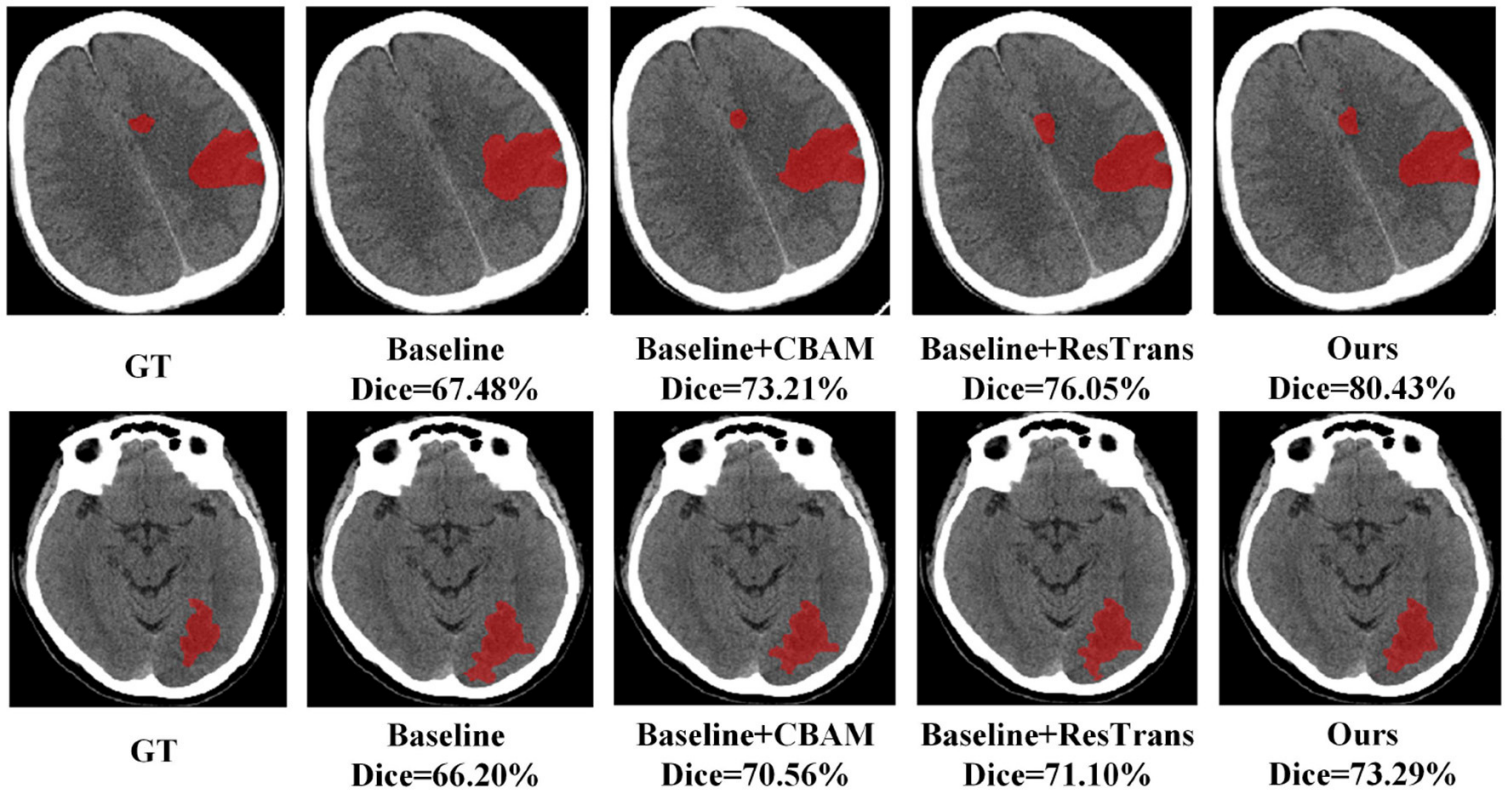
Two segmentation examples obtained in the ablation study.

In this study, CBAM was added to direct attention to the CNN Encoder and a residual connection was used on the Transformer encoder to enhance the detail features. To prove the effectiveness of these two methods, we conducted baseline experiments on the method with only CBAM added and the method with only residual structure added. As shown in Table 6, both structures enhance the segmentation efficiency of the baseline method, and the integration of these two operations can achieve better results. Two segmentation examples of each method and the Dice score in the AISD infarct segmentation task are shown in Figure 5.

**Table 6:** Ablation study results of our method for the AISD dataset.

**Method**	**Dice (%)**	**F1 score**	**Recall**	**Precision**
Baseline	55.57	0.6024	0.6099	0.5951
Baseline+CBAM	56.13	0.6101	0.6273	0.5939
Baseline+ResTrans	56.75	0.6120	0.6313	0.5940
**Ours**	**58.66**	**0.6298**	**0.6319**	**0.6278**

The bold values indicate the optimal performance of the metric in the experiment.

## 4. Discussion

In the AIS infarct segmentation task of AISD, our proposed method achieved a Dice score of 58.66%, surpassing previous methods. The experimental results show that the fusion framework of CNN and Transformer effectively captured both local and global information from NCCT scans. Ablation studies highlight the contributions of CBAM and the residual structure of the Transformer Encoder improving segmentation efficiency. Volumetric analysis reveal a strong correlation between our method and manually segmented infarcts. Furthermore, our method achieves the best performance in a dichotomization analysis, with a cut-off value of 70 cc, demonstrating its accuracy in classifying infarct volumes. These results indicate the potential of our method to provide valuable infarct information for clinical diagnosis and practice.

## 5. Conclusion

To achieve automatic and accurate segmentation of AIS infarcts, we proposed a segmentation method in this study based on the convolutional attention mechanism and Deformable Transformer. Our method used a CNN Encoder to extract these features, augmented by the CBAM to enhance the importance of these features. Additionally, a Deformable Transformer Encoder is used to model the global dependencies and reduce complexity. We incorporated residual connections before and after the Transformer Encoder to enhance the local features. The segmentation results for AISD demonstrates the superior performance of our method, offering a novel solution for AIS infarct segmentation and improving segmentation accuracy.

## Data availability statement

Publicly available datasets were analyzed in this study. This data can be found at: GitHub, https://github.com/GriffinLiang/AISD.

## Ethics statement

Ethical review and approval was not required for the study on human participants in accordance with the local legislation and institutional requirements. Written informed consent from the patients/participants or patients/participants' legal guardian/next of kin was not required to participate in this study in accordance with the national legislation and the institutional requirements.

## Author contributions

ZX: methodology, formal analysis, visualization, and writing—review and editing. CD: conceptualization, review and editing, and funding acquisition. Both authors contributed to the article and approved the submitted version.
